# The impact of mental health disorders and job demands on the individual job performance of construction workers

**DOI:** 10.1093/joccuh/uiae060

**Published:** 2024-10-08

**Authors:** Gokhan Kazar, Pourya Rahmanzadeh

**Affiliations:** Department of Civil Engineering, Istanbul Medeniyet University, 34700, Istanbul, Turkey; Department of Civil Engineering, Istanbul Gelisim University, 34310, Istanbul, Turkey; Department of Civil Engineering, Yildiz Technical University, Istanbul, Turkey; Graduate Student, Department of Civil Engineering, Istanbul Gelisim University, Istanbul 34310, Turkey

**Keywords:** mental health disorders, worker productivity, job demands, structural equation modeling, interaction effect, demographic characteristics

## Abstract

**Objectives:**

Understanding the impact of job demands and mental health disorders on individual job performance is crucial to achieving a more productive workforce and should be empirically investigated. Therefore, the main purpose of this study was to assess the impact of job demands and mental health disorders on individual job performance among construction workers. In addition, we examined the interaction effect between job demands and some demographic characteristics (income, marital status, experience, and area of residence) on the job performance of construction workers in 2 dimensions.

**Methods:**

For this purpose, a new conceptual model and 3 different hypotheses were introduced. A survey was designed and administered to 513 construction workers. Whereas the measurement items regarding demographic characteristics, job demands, and mental health disorders were addressed to construction workers, the last part related to job performance of construction workers was conducted with site managers of the workers to obtain more objective results. A structural equation modeling approach was adopted to assess the validity of the model and to test the hypotheses.

**Results:**

The results of this study show that whereas job demands have a significant and high impact on individual job performance, the effect of mental health disorders on job performance is significant at a moderate level. In addition, the demographic characteristics of marital status and area of residence have a significant interaction effect on job performance when combined with job demands.

**Conclusion:**

Providing individualized support systems, resources, and opportunities for construction workers can help mitigate the negative effects of excessive demands and mental disorders on labor productivity.

## Teaser

Key points

- **What is already known on this topic:** Construction workplaces are harsh working environments, resulting in high job demands and mental disorders for construction workers. These problems could have a detrimental effect on the work performance of construction workers. However, the impact of these problems on construction workers is still unclear and should be empirically investigated. In addition, the interaction effect of these factors with some demographic characteristics is not known.
- **What this study adds:** We developed a conceptual model and relevant hypotheses to understand the impact of job demands and mental health disorders on individual job performance of construction workers. The results of this study will provide new insights into this concept. Understanding the interaction effect between job demands and demographic characteristics is also a new contribution to the body of knowledge.
- **How this study might affect research, practice, or policy:** By understanding the interplay between job demands, mental health disorders, and worker outcomes, organizations can implement strategies to optimize productivity while protecting the health and well-being of construction workers. Providing individualized support systems, resources, and opportunities for construction workers can help mitigate the negative effects of excessive demands and mental disorders on labor productivity.

## 1. Introduction

Productivity challenges in the construction industry are complex and can be attributed to a number of factors. Research has shown that low productivity in construction projects is often related to issues such as low labor productivity, especially in developing countries where construction activities are labor intensive.^1^ Factors that affect construction productivity include the availability of materials, tools, rework, equipment, and motivational dynamics of workers.^2^ Rework in construction projects has also been identified as a major factor affecting productivity and workflow within the construction supply chain.^3^

One of the main issues behind the productivity problems in the construction industry is poor labor productivity and shortage of skilled labor. Labor productivity in the construction industry is influenced by various factors that can affect the efficiency and effectiveness of labor in construction projects. Enshassi et al^4^ identified challenges such as difficulty in recruiting supervisors and workers, high labor turnover, absenteeism, and communication problems with foreign workers as significant barriers to productivity. Ghoddousi and Hosseini^2^ identified factors such as shortage of materials, lack of experienced workers, misunderstandings between workers and supervisors, delays in payment, and lack of equipment as major contributors to reduced labor productivity in construction projects. In addition, Mahamid^5^ highlighted factors such as rework, lack of cooperation, owner’s financial status, lack of labor experience, and material shortages as major issues that negatively affect labor productivity in building construction.

In addition, job demands could have a significant impact on the productivity of construction workers. Job demands have a significant impact on construction workers, affecting various aspects of their well-being and work productivity. Job demands refer to the physical, psychological, social, or organizational aspects of a job that require sustained effort or skill on the part of an employee and are associated with certain physiological and psychological costs. In simpler terms, job demands are those elements of a job that require effort from the employee and can lead to stress or strain, especially when demands are high and resources or support are low. The job demands–resources (JD-R) model suggests that job demands, such as workload and job strain, can lead to negative outcomes such as burnout and mental health problems.^6^ Studies have also shown that job demands can lead to mental ill health and low motivation level in construction workers.^7^ Research has also shown that high workload, physical and cognitive demands, and work intensity can negatively affect the well-being and mental health of construction workers.^8^^,^^9^ In the construction industry, job demands can vary considerably depending on the specific role, project requirements, and working environment. In construction projects there are some common job demands, such as physical demands, workplace hazards, time pressure, team coordination, adaptability to change, and skill requirements.

Construction work often involves manual labor and physical exertion. Workers may be required to lift heavy objects, operate machinery and equipment, work in awkward positions, or perform tasks in varying weather conditions. The physical demands of construction work can lead to fatigue, musculoskeletal injuries, and physical stress.^9^ In addition, construction sites can be inherently dangerous environments with risks such as falls, exposure to hazardous materials, electrical hazards, and accidents involving heavy machinery. Construction projects often have tight deadlines and schedules to meet client expectations and contractual obligations. Workers may be under pressure to complete tasks within specified timeframes, which can contribute to stress and affect productivity.^10^ The changing environment of construction projects, due to design changes, supply chain disruptions, or unforeseen site conditions, is another common job demand that affects workers’ adaptability and productivity.^11^

It is important to consider both job demands and mental disorders when creating a work environment conducive for construction workers to promote their health and well-being. It is also necessary to incorporate job-specific demands and health effects into workplace health and safety practices to ensure the well-being of construction workers and enhance their individual job performance.^6^^,^^12^ However, to the authors’ knowledge, no study has investigated the impact of job demands and mental health disorders on the individual job performance of construction workers. Therefore, the main aim of this study was to understand the relationship between job demands, mental health disorders, and the impact of these factors on the individual job performance of construction workers. In addition, we assessed the interaction effect of job demands and demographic characteristics on the job performance of construction workers. Maintaining worker well-being, productivity, and overall organizational performance requires a balance between job demands and job performance of construction workers. By understanding the interplay between job demands, mental health disorders, and worker outcomes, organizations can implement strategies to optimize productivity while protecting the health and well-being of construction workers. Providing individualized support systems, resources, and opportunities for construction workers can help mitigate the negative effects of excessive demands and mental disorders on labor productivity.

## 2. Methods

### 2.1. Conceptual model and hypotheses development

**Figure 1:**
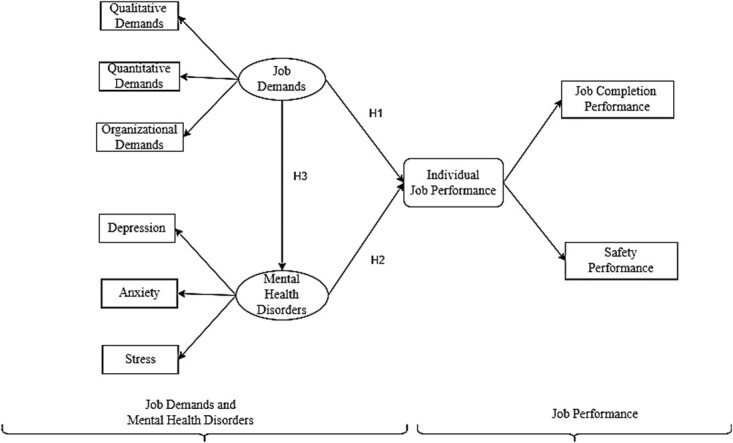
Conceptual model.

Cottini and Lucifora^14^ found that there could be a strong relationship between adverse working conditions, such as job demands and hazards, and mental health problems among workers. Moreover, excessive job demands can lead to job dissatisfaction, stress, and burnout, all of which can impact retention and productivity of construction workers.^13^ Hynek et al^16^ highlighted the link between mental disorders and reduced work-related productivity and earnings, particularly the negative impact on employment. This link between mental disorders and labor force outcomes is crucial for understanding the wider impact of mental health on productivity. Based on previous studies that highlight the potential impact of job demands and mental health disorders on job performance of construction workers, we developed our main 3 hypotheses and relevant conceptual model (Figure 1).

**H1**: Job demands in construction workplaces have a significant impact on individual job performance of construction workers.

**H2**: Mental health disorders (eg, depression, anxiety, and stress) have a significant impact on individual job performance of construction workers.

**H3**: Job demands in construction workplaces have a significant impact on the mental health disorders of construction workers.

In addition to testing the main hypotheses, we evaluated the interaction effect of some important demographic characteristics with job demands. As some previous studies have highlighted the role of demographic characteristics on employees’ job performance, we assessed the interaction effects of some important demographic characteristics such as marital status, experience, area of residence, income, and education level with job demands on construction workers’ individual job performance.^15^ In other words, we assessed how the impact of job demands on job performance varied according to demographic characteristics.

### 2.2. Research methodology

**Figure 2:**
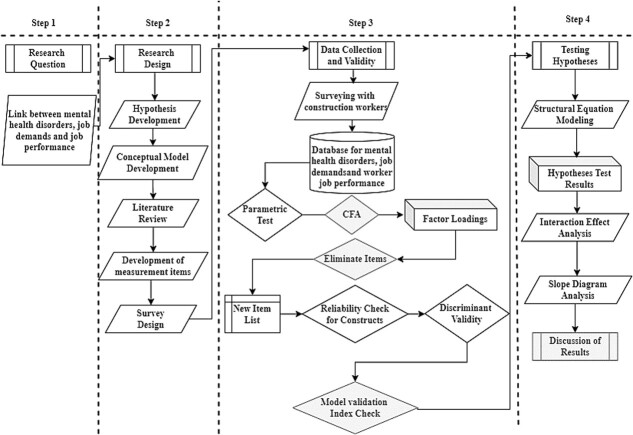
Research methodology.

We developed the conceptual model and main hypotheses after identifying the research gap and our primary research questions. A thorough review of the literature was carried out in order to construct a survey based on the developed hypotheses and conceptual model. The necessary data were then collected using a face-to-face survey approach. In order to determine the factor loading of each measure, the data were first examined for normal distribution after data collection. Next, a number of indices were used to assess the reliability of the proposed model. Finally, structural equation modeling (SEM) was employed to test our primary theories, and slope diagrams were used to evaluate the interaction effects of job demands and demographic characteristics on the job performance of construction workers (Figure 2).

### 2.3. Data collection and analysis

We used a self-administered survey approach to assess the reliability of the conceptual model and hypotheses. The survey is divided into 4 sections: (1) participant demographics, (2) assessment of mental disorder level, (3) job demands, and (4) individual job performance of construction workers. The first section of the survey asked questions about age, work experience, marital status, place of residence, tobacco use, number of hours worked per week, and monthly income. The Depression, Anxiety, and Stress Scale (DASS-21), a mental health assessment instrument with 21 questions—7 for each disorder—was used for the second section. The DASS-21, developed by Lovibond and Lovibond,^33^ is a commonly used research tool for assessing the severity of mental health disorders. In the third part, we asked about the job demands such as quantitative (3 measurements), qualitative (4 measurements), and organizational demands (5 measurements) with a total of 12 different measurement items. The questions in this section related to job demands were drawn from previous studies^17^^-^^19^ that used the JD-R model and were added to the questionnaire according to the purpose of the study. In the last section, 2 different job performance categories, such as job completion and job safety, were developed with 5 and 4 items, respectively, and it was investigated how and to what extent the other measured parameters (job demands and mental health disorders) directly affect the individual job performance of construction workers. For the current study these questions were also taken and adopted from previous research.^19^^,^^20^ Whereas the first 3 parts were conducted with construction workers, responses for the final part about individual job performance of construction workers were collected from the site chief for the corresponding construction worker(s) to achieve more objective results. Likert scales, where 1 is “strongly disagree” and 5 is “strongly agree,” were used to rate each item on mental health disorder, job demand, and the job performance of construction workers.

The University Ethics Committee provided the necessary ethical approval prior to collecting the survey data. We gave a brief summary of the research project at the beginning of the survey and assured respondents that no personal information would be collected or stored and that all data would be used for research purposes only.

We conducted a path analysis using an SEM approach to test the hypotheses of the study. SEM allows researchers to simultaneously examine multiple relationships among variables within a single model. This capability is particularly valuable when studying complex phenomena with interrelated constructs, such as the relationships between latent constructs (eg, attitudes, personality traits) and observed variables (eg, survey responses). Researchers can specify a hypothesized model and evaluate its fit to the data using statistical indices such as c^2^ tests, comparative fit indices (CFIs), and root mean square error of approximation (RMSEA). This process helps researchers assess the degree to which their models accurately represent the relationships among variables. By doing so, SEM provides more accurate estimates of the relationships between latent constructs and reduces bias in parameter estimates. Overall, SEM offers a powerful framework for analyzing complex relationships among variables, providing researchers with a versatile tool for theory testing, model evaluation, and hypothesis generation in various fields of research. We also used slope analysis diagrams to evaluate the interaction effects of job demands and demographic characteristics on the individual job performance of construction workers.

## 3. Results

In order to evaluate the conceptual model and test the hypotheses, a total of 513 construction workers from 93 different Turkish and Iranian construction companies participated in the study. For worker evaluation, 113 construction professionals participated in this study. The majority of the data were collected for 3 different types of projects: infrastructure (25.34%), transportation (28.65%), and buildings (46%).

### 3.1. Validity and reliability of conceptual model

The validity and reliability of the measures of job demands, mental health disorders, and individual job performance of construction workers were assessed prior to testing the developed hypotheses. First, the normal distribution of each latent variable group was examined using skewness and kurtosis values. The results show that the dataset had a normal distribution,^21^ as all the skewness values of the measures fell between −1 and +1, and their kurtosis values fell between −3 and +3. The unidimensionality and convergent validity of the latent variables were then tested using confirmatory factor analysis (CFA). In order to achieve and evaluate the factor loadings, we used IBM SPSS statistical software 25.0 version. Prior to testing our hypotheses, items H-D1, H-A3, H-A7, H-S3, JD-QL1, JD-O5, and JC3 were removed based on CFA because their factor loadings were less than 0.6 and not statistically significant (*P* > .05).^22^ Thus, our aim was to improve the internal consistency and reliability of the dataset (Table 1).

**Table 1:** Validity and reliability results of conceptual model.

**Main constructs**	**Latent construct**	**Observed item**	**Mean**	**Factor loading**	**Cronbach** $ \alpha $	**Composite reliability**	**GFI**	**RMSEA**	**χ** ^**2**^ **/*df***	**AVE**
**Mental health disorders**	Depression	H-D1	2.568	Deleted	.876	0.911	0.938	0.013	0.926	0.692
		H-D2		0.759						
		H-D3		0.842						
		H-D4		0.628						
		H-D5		0.786						
		H-D6		0.774						
		H-D7		0.763						
	Anxiety	H-A1	2.123	0.851						
		H-A2		0.648						
		H-A3		Deleted						
		H-A4		0.589						
		H-A5		0.782						
		H-A6		0.658						
		H-A7		Deleted						
	Stress	H-S1	2.782	0.814						
		H-S2		0.903						
		H-S3		Deleted						
		H-S4		0.741						
		H-S5		0.658						
		H-S6		0.713						
		H-S7		0.641						
**Job demands**	Quantitative	JD-QN1	2.952	0.781	.812	0.789	0.974	<0.001	0.851	0.718
		JD-QN2		0.654						
		JD-QN3		0.812						
	Qualitative	JD-QL1		Deleted						
		JD-QL2		0.781						
		JD-QL3		0.635						
		JD-QL4		0.643						
	Organizational	JD-O1		0.746						
		JD-O2		0.569						
		JD-O3		0.603						
		JD-O4		0.672						
		JD-O5		Deleted						
**Individual job performance**	Job completion	JC1	2.861	0.782	.871	0.894	0.951	0.018	0.973	0.745
		JC2		0.624						
		JC3		Deleted						
		JC4		0.674						
		JC5		0.826						
	Safety performance	SP1	2.128	0.813						
		SP2		0.763						
		SP3		0.754						
		SP4		0.589						

Abbreviations: AVE, average variance extracted; GFI, goodness of fit index; RMSEA, root mean square error of approximation; H-D, depression; H-A, anxiety; H-S, stress; JD-QN, job demand-quantitative; JD-QL, job demand-qualitative; JD-O, job demand-organizational; JC, job completion; SP, safety performance.

Then, since Cronbach a coefficient and composite reliability (CR) are measures of internal reliability and consistency, respectively, they were used to assess reliability^21^. According to Fornell and Larcker’s^23^ study, the results show that the satisfactory level of latent constructs is indicated by Cronbach a coefficients and CR values greater than 0.7 for job demands (α = .812, CR = 0.789), mental health disorders (α = .876, CR = 0.911), and individual job performance (α = .871, CR = 0.894). We evaluated the RMSEA, the goodness of fit index (GFI), and the χ^2^ value based on the ratio χ^2^/*df* to determine the fitness of each latent model. Based on GFI, RMSEA, and χ^2^/*df*, the results show that each latent construct met the required level of reliability. For example, the latent construct job demands, with GFI = 0.974 greater than 0.9 and RMSEA <0.001 less than 0.1, met the requirements for model fit. In addition, we found that for the job demands construct χ^2^/*df* = 0.851 was less than 3^22^. SEM procedures to test the conceptual model and hypotheses were carried out using AMOS software.

The absolute correlation coefficient between each latent construct and the square root of the average variance extracted (AVE) for each construct must be less than 1 for discriminant validity to be met.^23^ The results indicate that each latent construct met the requirements for discriminant validity based on the square root of the AVE for mental disorders (0.831), job demands (0.847), and job performance (0.863) as well as the absolute correlation coefficients (mental disorders/job demand, ρ = 0.532; mental disorders/job performance, ρ = 0.351; job demands/job performance, ρ = 0.623).

Finally, the model fit of the proposed conceptual framework was assessed using a number of indices, including the goodness of fit index (GFI), the incremental fit index (IFI), the Tucker-Lewis index (TLI), the comparative fit index (CFI), the RMSEA, and the ratio χ^2^/*df*. According to Ketchen (2013)^22^, all goodness-of-fit indices (χ^2^/*df* = 1.218, IFI = 0.923, CFI = 0.917, TLI = 0.908, and RMSEA = 0.027) fall within the acceptable range and are sufficient for model fit.

### 3.2. Hypotheses test results

Next, we conducted a path analysis using SEM to test the proposed hypotheses. The SEM result indicated that job demands (β = −.828, *P* < .05) and mental health disorders (β = −.389, *P* < .05) have a significant and detrimental effect on individual job performance of construction workers. In addition, this study found a significant and positive effect of job demands on mental disorders (β = .694, *P* < .001). We can now accept our primary hypotheses H1, H2, and H3. Whereas the effect of job demands on mental disorders and individual job performance of construction workers is high (β > .5), the effect of mental health disorders on the individual job performance is moderate (.3 < β < .5).^16^

**Figure 3:**
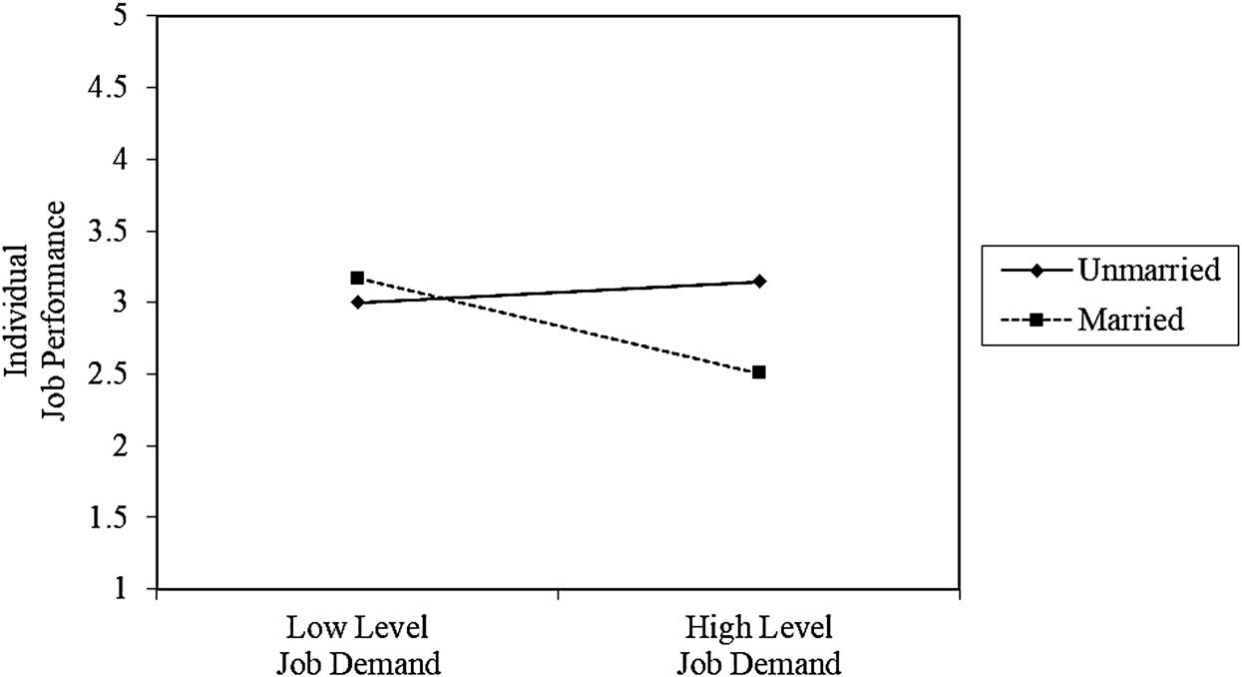
Interaction effect between marital status and job demands on job performance.

**Figure 4:**
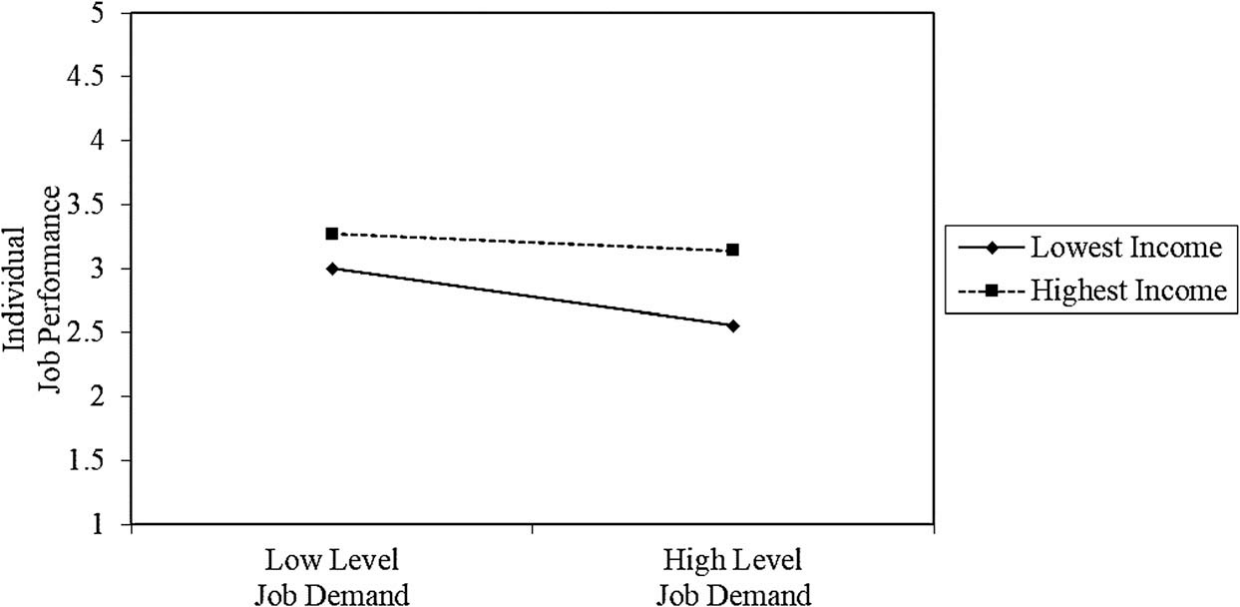
Interaction effect between income and job demands on job performance.

**Figure 5:**
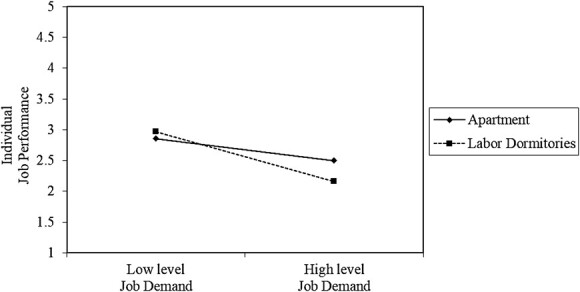
Interaction effect between area of residence and job demands on job performance.

### 3.3. Slope diagram results

The conclusion of the slope analysis indicated that when married construction workers experience high levels of job demands, the job performance of these construction workers significantly reduces. Here, the interaction effect between job demands and marital status on the job performance was observed for married workers (Figure 3). The result of the slope analysis also proved that construction workers with the lowest and highest incomes tend to have similar levels of job demands and the individual job performance of these 2 groups of construction workers slightly reduces. At this point, we can say that there is no significant interaction effect between income level and job demands on job performance (Figure 4). According to the result of the slope diagram, there was a significant interaction effect between area of residence and job demands on job performance for construction workers living in labor dormitories. In other words, the workers who live in labor dormitories tend to be more susceptible to high job demands, and thus their work productivity significantly reduces (Figure 5). On the other hand, no significant interaction effect was found between experience and job demands on employee productivity, as each group showed a similar trend in job performance when experiencing high levels of job demands.

## 4. Discussion

The SEM results show that the first main hypothesis, H1, which states that job demands have an impact on individual job performance of construction workers, is supported. This point is also well documented in the body of knowledge. Several studies have highlighted the negative effects of high job demands on employee performance.^24^^,^^25^ When employees are overwhelmed by the demands of their jobs, they can experience physical and emotional exhaustion, leading to decreased motivation and productivity.^14^ Excessive job demands can make it difficult for employees to focus and concentrate on their tasks. Constant interruptions, multitasking, and shifting priorities can impair cognitive function and hinder productivity.

The results of this study also show that there is a moderately significant effect of mental health disorders on individual job performance of construction workers. Mental health problems are prevalent in the working population and have various implications for workers, organizations, and workplace health.^9^ In addition, psychological distress can lead to poor job performance and significant mental health problems for some employees.^26^^,^^27^ Poor mental health, including anxiety, depressive symptoms, and job stress, has been found to negatively affect job performance.^27^^,^^28^ Construction work requires careful attention to detail and adherence to safety protocols, so any decrease in concentration can lead to errors, accidents, and rework, ultimately reducing productivity.^29^ Construction work often involves long hours and physically demanding tasks, so workers struggling with mental health issues may experience fatigue and reduced stamina, leading to lower productivity levels. Poor mental health can increase the risk of accidents and injuries on construction sites.^30^

The impact of job demands on mental health disorders was also found to be highly significant. Several studies have highlighted the negative association between job demands and mental health, with job demands being associated with increased mental strain and health symptoms.^9^^,^^26^^,^^29^ An imbalance between work demands and personal life can contribute to stress and strain on mental health. When work demands encroach on personal time and activities, individuals may experience feelings of overwhelm, guilt, and dissatisfaction, which can negatively affect their mental well-being.^25^

The relationship between job demands and mental disorders in construction is characterized by a strong connection between the high physical and psychological demands of construction work and an increased risk of mental health issues among workers. Construction workers face physically taxing tasks, such as heavy lifting, repetitive motions, and prolonged exposure to harsh environments (eg, extreme temperatures, noise, and hazardous materials).^27^ Workers often encounter stress from tight deadlines, long hours, shift work, and high job insecurity. They may also face high-pressure situations, such as working at heights or with dangerous machinery. The high-pressure environment, combined with the potential for injury and long working hours, can lead to depressive symptoms and anxiety disorders. The construction industry has higher rates of substance abuse, often used by workers as a coping mechanism to deal with stress or pain.^31^

The indirect pathway from job demands to individual job performance through mental health disorders highlights how high job demands can negatively affect an employee’s mental health, which in turn affects their job performance. This pathway is important to understand because it illustrates that the relationship between job demands and job performance is not always direct, but is often mediated by mental health outcomes. When employees face high job demands (eg, heavy workloads, tight deadlines, complex tasks, emotional strain) this can lead to significant stress. Prolonged exposure to these demands without adequate job resources can lead to psychological distress and mental health problems. The stress of high job demands can lead to anxiety or depressive symptoms. Workers may feel overwhelmed, have difficulty concentrating, and lose motivation. Mental health problems such as anxiety or depression can affect cognitive functions such as memory, decision-making, concentration, and problem-solving, leading to reduced productivity. Workers experiencing mental health problems may feel less motivated or engaged, resulting in lower quality work, less initiative, and poorer attention to detail. Research shows that burnout, a common outcome of high job demands, often mediates the relationship between job demands and job performance. High demands cause burnout, which then leads to a decline in job performance.^8^^,^^9^ Studies have also indicated that anxiety, depression, and other mental health disorders serve as mediators between job demands and job performance.^16^^,^^31^ For example, employees who experience high job demands may develop depressive symptoms that negatively affect their job performance by reducing their motivation, cognitive capacity, and resilience.

There is also a significant interaction effect between marital status and job demands; and between area of residence and job demands on individual job performance. An interaction effect between marital status and job demands on job performance suggests that the relationship between job demands and job performance varies by marital status. The interaction effect of marital status and job demands on job performance refers to how the combination of being married or single interacts with the level of job demands to influence an individual’s job performance. In this study we found that married workers are more prone to high job demands, and thus their individual performance significantly reduces. For example, it is possible that married people have additional responsibilities and worries about the future, which could exacerbate the effects of high job demands on job performance. This conflict may lead to distraction, absenteeism, or reduced productivity at work, thereby negatively affecting job performance.^29^

Individuals living in labor dormitories, which often have basic living conditions and limited amenities, may have fewer resources to cope with high job demands than those living in housing. The combination of challenging living conditions and demanding work requirements can create additional stressors that may lead to reduced job performance. The lack of privacy, comfort, and support networks in work camps may exacerbate the negative effects of high job demands on job performance. Individuals living in housing may have access to better living conditions, amenities, and support networks than those in labor dormitories. In this case, the negative impact of high job demands on job performance may be mitigated by the relatively more favorable living conditions provided by housing.^32^

## 5. Conclusion

The current study attempts to examine the impact of job demands and mental health disorders on the individual job performance of construction workers. To this end, we first developed a conceptual model and hypotheses. We also assessed this impact in relation to some important demographic characteristics. In this context, a survey was designed and data were collected from 513 construction workers in Iran and Turkey. An SEM approach was used to understand the relationship between job demands, mental health disorders, and job performance of construction workers. The results show that both mental health disorders and job demands have significant negative influence on job performance of construction workers. Also, marital status and area of residence have a significant interaction effect with job demands on job performance. To the best of the authors’ knowledge, these points are evaluated and highlighted for the first time in the body of knowledge.

One of the most important theoretical contributions of this study is to provide an understanding of poor job performance of construction workers in terms of job demands and mental health disorders. In addition, this study assessed the impact of job demands on construction workers’ mental health disorders. We also examined the interaction effect between job demands and demographic characteristics on the job performance of construction workers. By doing so, we can interpret how job demands influence construction workers’ job performance in terms of demographic characteristics. This approach will open up new future research directions to understand poor labor productivity.

Although we believe that the current research will make significant contributions, it has some limitations. The data were collected in only 2 different developing countries. The results may change for developed and underdeveloped countries, and it is not possible to generalize the results to the entire construction industry. This study did not consider other psychological parameters such as burnout, work–family conflict, and job satisfaction and the effectiveness of coping strategies against these issues. For the JD-R model, we only included the effects of job demands on individual job performance of construction workers. Job resources also can be included in the model for future studies. Moreover, since the job performance of construction workers was rated by the supervisors at each site, the data have a hierarchical structure. Thus, the data consist of relative evaluations within each site, which are difficult to compare over sites. This aspect could heavily influence the final results. All these limitations in this study can be addressed in future research with different approaches.

We believe that the results of this study shed light on workforce productivity issues by providing relevant information on the causes related to job demands and mental health disorders. Construction companies can take several steps to address mental health disorders and reduce job demands on construction sites to increase the well-being and productivity of construction workers. Construction companies can create an environment where employees feel comfortable discussing their mental health concerns without fear of stigma or discrimination. Construction companies could regularly solicit feedback from employees about their experiences and concerns related to mental health and job demands. All of these proactive practices will result in a healthier, more productive, and sustainable construction work environment.

## Supplementary Material

Supplemantary_File_uiae060

## Data Availability

Some or all data, models, or code that support the findings of this study are available from the corresponding author upon reasonable request.
