# Supplementary material for: The impact of mental health disorders and job demands on the individual job performance of construction workers
Source: J Occup Health. 2024 Oct 8;66(1):uiae060. doi: 10.1093/joccuh/uiae060 (PMC11520402; doi:10.1093/joccuh/uiae060)
Supplement: Supplemantary_File_uiae060 [file supplemantary_file_uiae060.docx]

**Supplemantary File**

**Descriptive statistics for respondents**

| Demographic Characteristics | Subcategory | Frequency | Percentage (100%) |
| --- | --- | --- | --- |
| Marital status | Married | 254 | 49.51 |
|  | Unmarried | 259 | 50.49 |
| Tobacco usage | Yes | 276 | 53.80 |
|  | No | 237 | 46.20 |
| Weekly working hour | 40-45 | 27 | 5.26 |
|  | 45-50 | 83 | 16.18 |
|  | 50-55 | 251 | 48.93 |
|  | More than 55 | 152 | 29.63 |
| Living space | Apartment | 223 | 43.47 |
|  | Labor dormitories | 290 | 56.53 |
| Income (per month) | 300 - 500 $ | 56 | 10.92 |
|  | 500 - 750 $ | 197 | 38.40 |
|  | 750 - 1000 $ | 219 | 42.69 |
|  | More than 1000 $ | 41 | 7.99 |
| Work experience | 0-5 years | 110 | 21.44 |
|  | 5-10 years | 163 | 31.77 |
|  | 10-15 years | 144 | 28.07 |
|  | More than 15 years | 96 | 18.71 |
| Age | Between 20-30 | 192 | 37.43 |
|  | Between 30-40 | 179 | 34.89 |
|  | Between 40-50 | 104 | 20.27 |
|  | More than 50 | 38 | 7.41 |
| Project type | Building | 236 | 46.00 |
|  | Transportation | 147 | 28.65 |
|  | Infrastructure | 130 | 25.34 |
